# Two Genome Sequences of *Klebsiella pneumoniae* Strains with Sequence Type 23 and Capsular Serotype K1

**DOI:** 10.1128/genomeA.01097-16

**Published:** 2016-10-20

**Authors:** Hsi-Hsu Lin, Yao-Shen Chen, Hao-Wen Hsiao, Pei-Tan Hsueh, Wei-Fen Ni, Ya-Lei Chen

**Affiliations:** a Section of Infectious Disease, Department of Medicine, E-Da Hospital, Kaohsiung, Taiwan; b Department of Internal Medicine, E-Da University, Kaohsiung, Taiwan; c Division of Infectious Diseases, Kaohsiung Veterans General Hospital, Kaohsiung, Taiwan; d Department of Biotechnology, National Kaohsiung Normal University, Kaohsiung, Taiwan; e Department of Biological Sciences, National Sun Yat-sen University, Kaohsiung, Taiwan

## Abstract

Here, we report the whole-genome sequences of *Klebsiella pneumoniae* ED2 and ED23, isolated, respectively, from bacteremic patients with liver abscesses (ED2) and patients with primary liver abscess and metastatic meningitis (ED23). Both strains were of multilocus sequence type 23 with capsule serotype K1.

## GENOME ANNOUNCEMENT

*Klebsiella pneumoniae* is a Gram-negative, rod-shaped bacterium that causes community- and hospital-acquired infections (1, 2). Worldwide, community-acquired primary liver abscess caused by invasive *K. pneumoniae* with serotype K1 has emerged. In Taiwan, approximately 12% of patients with *K. pneumoniae* liver abscess appeared with a metastatic brain abscess or pyogenic meningitis (3). This unique epidemic of *K. pneumoniae*–caused meningitis has become a public health concern in Taiwan (4).

*K. pneumoniae* ED2 or ED23 strains were isolated, respectively, from bacteremic patients with liver abscesses and patients with liver abscesses and meningitis. Both strains were of multilocus sequence type 23 with a K1 capsular serotype.

Total DNA was extracted from both strains by a mini-QIAamp DNA isolation kit (Qiagen, Germany). The entire genomic sequence was determined using PacBio technologies (Pacific Biosciences, USA). Continuous long reads with a 20-kb average insert size were generated, and read processing and *de-novo* assembly were performed using the HGAP program version 3 (bioinformatics analysis served by Yourgene Biotech, Inc., New Taipei, Taiwan). A comparison of the ED2 and ED23 whole-genome sequences was performed using the MUMmer program version 3.22 (5).

The assembled genome sizes were 5,412,960 bp with a G+C composition of 57.4% for strain ED2 and 5,374,626 bp with a G+C composition of 57.6% for strain ED23. Strain ED23 harbored a plasmid of 212,770 bp with a G+C composition of 50.1%. The similarity in the chromosomal DNA sequences of both strains was 99.93%. In total, there were 21 sites with gaps appearing between mutually consistent alignments, 11 sites appearing in sequence rearrangement, and 7 sites with inserted duplication.

### Accession number(s).

The whole-genome sequences of *K. pneumoniae* ED2 and ED23 have been deposited in GenBank under the accession numbers CP016813 (ED2, chromosome), CP016814 (ED23, chromosome), and CP016815 (ED23, plasmid).
